# *RAS* testing practices and *RAS* mutation prevalence among patients with metastatic colorectal cancer: results from a Europe-wide survey of pathology centres

**DOI:** 10.1186/s12885-016-2810-3

**Published:** 2016-10-26

**Authors:** Annemarie Boleij, Véronique Tack, Aliki Taylor, George Kafatos, Sophie Jenkins-Anderson, Lien Tembuyser, Els Dequeker, J. Han van Krieken

**Affiliations:** 1 Department of Pathology, Radboud University Medical Centre, Geert Grooteplein-Zuid 10, 6525 GA Nijmegen, The Netherlands; 2 Department of Public Health and Primary Care, University of Leuven, Herestraat 49, Box 602, 3000 Leuven, Belgium; 3 Centre for Observational Research, Amgen Ltd, 1 Uxbridge Business Park, Uxbridge, UB8 1DH UK; 4 Adelphi Research (Global), Adelphi Mill, Bollington, Manchester, SK10 5JB UK

**Keywords:** *RAS* testing, *KRAS*, *NRAS*, Prevalence, Laboratory practices, Metastatic colorectal cancer

## Abstract

**Background:**

Treatment options for patients with metastatic colorectal cancer (mCRC) include anti-epithelial growth factor therapies, which, in Europe, are indicated in patients with *RAS* wild-type tumours only and require prior mutation testing of “hot-spot” codons in exons 2, 3 and 4 of *KRAS* and *NRAS*. The aim of this study was to evaluate the implementation of *RAS* testing methods and estimate the *RAS* mutation prevalence in mCRC patients.

**Methods:**

Overall, 194 pathology laboratories were invited to complete an online survey. Participating laboratories were asked to provide information on their testing practices and aggregated *RAS* mutation data from 20 to 30 recently tested patients with mCRC.

**Results:**

A total of 96 (49.5 %) laboratories across 24 European countries completed the survey. All participants tested *KRAS* exon 2, codons 12 and 13. Seventy (72.9 %) laboratories reported complete testing of all *RAS* hot-spot codons, and three (3.1 %) reported only testing *KRAS* exon 2. Sixty-nine (71.9 %) laboratories reported testing >80 patients yearly for *RAS* mutation status. Testing was typically performed within the reporting institution (93.8 %, *n* = 90), at the request of a treating oncologist (89.5 %, *n* = 85); testing methodology varied by laboratory and by individual codon tested. For laboratory *RAS* testing, turnaround times were ≤10 working days for the majority of institutions (90.6 %, *n* = 87). The overall crude *RAS* mutation prevalence was 48.5 % (95 % confidence interval: 46.4–50.6) for laboratories testing all *RAS* hot-spot codons. Prevalence estimates varied significantly by primary tumour location, approximate number of patients tested yearly and indication given for *RAS* testing.

**Conclusion:**

Our findings indicate a rapid uptake of *RAS* testing in the majority of European pathology laboratories.

## Background

In recent decades, changing clinical practices, in conjunction with the introduction of novel therapeutic agents, have resulted in improved outcomes for patients with metastatic colorectal cancer (mCRC) [1, 2]. Despite this, the worldwide burden represented by colorectal cancer (CRC), both in terms of incidence and mortality, remains substantial [3, 4]. In Europe, CRC is now the second most common malignancy. In 2012, approximately 447,000 new cases of CRC were diagnosed, with an estimated 215,000 CRC-related deaths, representing 11.6 and 13.0 % of all cancer-related deaths in men and women, respectively [5]. Approximately 20–25 % of patients with CRC will have evidence of metastatic disease at the time of their diagnosis, and a further 40–50 % of all patients with CRC will eventually develop metastases during the course of their illness [6, 7].

Monoclonal antibody (mAb) therapies that target the epidermal growth factor receptor (EGFR), such as cetuximab and panitumumab, have been shown to improve survival in patients with mCRC, both as monotherapies and in combination with conventional chemotherapy regimens [8–11]. Anti-EGFR mAbs have been found to be ineffective in CRC patients with mutations affecting the rat sarcoma viral oncogene homolog (*RAS*) gene family, which includes the kirsten *RAS* (*KRAS*) and neuroblastoma *RAS* (*NRAS*) oncogenes [10, 12, 13]. Mutations affecting specific codons (so-called “hot-spot” codons) in exons 2, 3 and 4 of the *KRAS* and *NRAS* genes have been identified, which predict non-response to anti-EGFR mAbs and allow the further malignant proliferation of tumour cells, despite treatment [10, 14].

Initial research focused primarily on mutations of *KRAS* exon 2, codons 12 and 13, which were originally found to predict resistance to cetuximab and panitumumab [13–15]. This led major oncology societies to recommend that *KRAS* exon 2 mutation status should be determined prior to anti-EGFR treatment [16, 17]. Therefore, treatment with anti-EGFR mAbs previously only required confirmation of *KRAS* wild-type status; however, in 2013, the European Medicines Agency (EMA) revised the therapeutic indication, restricting it to patients with *RAS* wild-type mCRC tumours only. Consequently, testing of hot-spot codons in exons 2, 3 and 4 of *KRAS* and *NRAS* is now a requirement prior to initiating treatment [18, 19]. This change was made in response to growing evidence of the effects of *RAS* family mutations in CRC. Key findings included efficacy analyses of first-line anti-EGFR therapy, in combination with chemotherapy, by *RAS* mutation status, which demonstrated that additional *RAS* mutations (other than *KRAS* exon 2) were predictive biomarkers for non-response to treatment [10].

The revised EMA indication for the use of anti-EGFR therapies highlights the need for consistent testing of the *RAS* mutation status of patients with mCRC prior to commencing treatment. The main aim of this retrospective survey was to assess the implementation of *RAS* testing in Europe and to investigate whether there is any variation in laboratory testing practices and turnaround times. An additional aim was to estimate the *RAS* mutation prevalence in patients with mCRC, according to predefined clinical and demographic characteristics.

## Methods

### Participating institutions

Pathology laboratories from 26 European countries currently or recently participating in the ongoing external quality assurance (EQA) scheme of the European Society of Pathology (ESP) for the testing of *RAS* mutations in CRC were invited to take part in this study. For each laboratory, a molecular biologist, pathologist or other laboratory representative (e.g. technician) was contacted directly by the study investigators and supplied with a unique survey link in order to allow online completion of the survey questionnaire and data collection form.

### Survey composition and variables

The online survey was divided into two parts. The first part included general questions about the characteristics of the participating laboratory, clinical indications for *RAS* mutation testing, DNA extraction method used and *RAS* mutation testing methods for each codon tested. In the second part of the survey, the participating laboratory was requested to provide aggregated data from approximately 20–30 of the most recent patients with mCRC tested for *RAS* mutation status. This section of the survey collected data on *RAS* mutation prevalence, including a breakdown by codon, the site of the patient’s primary tumour, the tissue sample site and the approximate turnaround time for *RAS* mutation testing. Turnaround time was defined as the time from receiving the request for *RAS* mutation testing to reporting of the result back to the requesting oncologist, grouped into 1–5, 6–10 and >10 working days.

The following codons were included in the online survey: *KRAS* and *NRAS* exon 2, codons 12 and 13; *KRAS* and *NRAS* exon 3, codons 59 and 61; and *KRAS* and *NRAS* exon 4, codons 117 and 146*.*

Prior to commencement of the study, the survey questions were tested on three pathologists/molecular biologists to assess the clarity of the survey questions and amended accordingly.

### Data collection

Survey results were collected in an anonymised fashion to ensure that it would not be possible to link answers to individual pathologists, molecular biologists or pathology centres. Collection of aggregated patient data from electronic pathology records ensured patient anonymity and therefore individual patient consent was not required. Each participating institution was assigned a unique identifying code and communication with the institutions was carried out by an independent third party. Non-responding institutions were identified via any unused identification codes; the third party at Radboud University Medical Centre reported these codes to investigators at the University of Leuven, who sent survey reminders to the institutions. Reminders were sent to non-responders 4 weeks after their initial invitation and again 2 weeks before the survey closed. Data checks were conducted daily during the data collection period to ensure data quality and address any data-related issues.

### Statistical analysis

A descriptive analysis of the laboratory characteristics and testing methods reported in the first part of the survey was carried out.

The overall *RAS* mutation prevalence and prevalence by patient characteristics and testing methods were calculated from the aggregated patient data reported in the second part of the survey. *RAS* mutation prevalence was calculated for all patients and for the subgroup of patients tested for all *RAS* hot-spot codons. The 95 % confidence interval (CI) was calculated for each prevalence result using the Clopper–Pearson exact method. Comparisons of *RAS* mutation prevalence according to laboratory and patient characteristics were made using the Pearson chi-squared test.

## Results

### Study participants

**Fig. 1:**
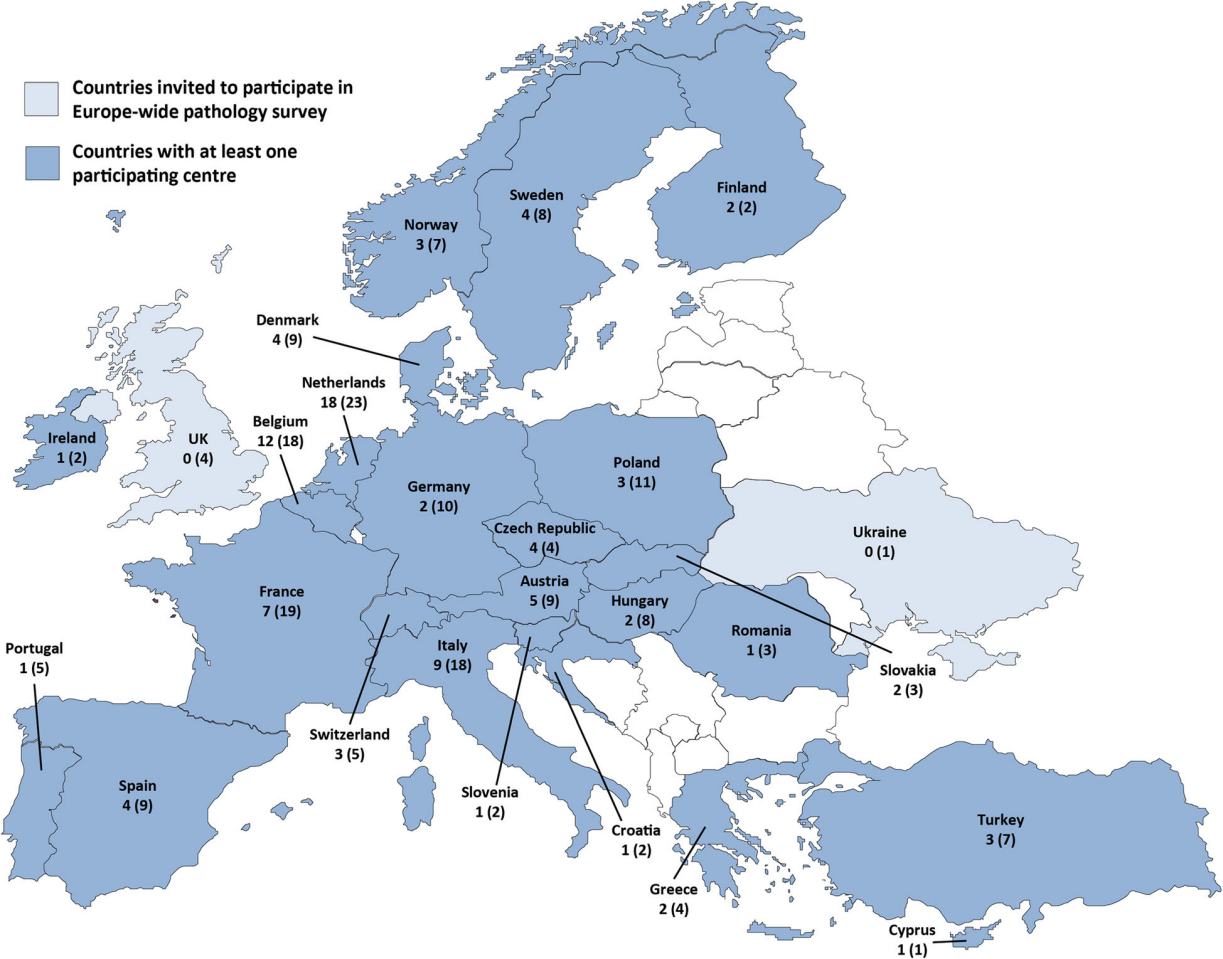
Survey responses by country, showing number of participating institutions and invited institutions

A total of 194 pathology laboratories at hospitals and institutions across 26 European countries were invited to participate in the survey. Of the institutions contacted, 96 (49.5 %) laboratories in 24 of the countries satisfactorily completed the online questionnaire between October and December 2014. The average positive response rate, by country, was 48.6 % of the invited laboratories with a largely even distribution throughout Europe (Fig. 1).

Of the laboratories invited to participate in the study, 63 were listed as accredited on the website of their national accreditation body (NAB). In each country the NAB is the organisation responsible for assessing adherence to laboratory standards issued by the independent International Organisation for Standardisation (e.g. CCKL in the Netherlands and Cofrac in France). In total, 43.8 % (*n* = 42) of the participating institutions were listed as accredited. Additionally institutions that were accredited were significantly more likely to respond to the survey; a 66.7 % (*n* = 42) positive response rate was obtained from the 63 accredited institutions, compared with a 41.2 % (*n* = 52) positive response rate from the 131 without NAB accreditation.

General hospitals and anti-cancer centres had a high positive response rate of 51.1 % (*n* = 46) as did universities and university hospitals (54.2 %, *n* = 39); these two broad categories made up the majority of the 96 respondents (47.9 % and 40.6 %, respectively). The remaining invited laboratories were listed as industry (*n* = 4) and private or private hospital (*n* = 28); these categories had numerically lower positive response rates, of 25.0 % (*n* = 1) and 35.7 % (*n* = 10), respectively, but given the low numbers of institutions in these categories this was not significantly different from the other categories. Invited institutions that had successfully passed their most recent ESP EQA scheme did not have significantly higher positive response rates than those institutions that had not passed (52.5 % and 34.4 %, respectively).

All 96 laboratories that responded completed the initial questionnaire part of the survey and 90 (93.8 %) of these respondents provided aggregated patient data in the second part of the survey. In total, aggregated data were collected from 3,259 patients with CRC, of whom the majority probably had metastatic disease. Of these 96 institutions, 71.9 % (*n* = 69) estimated that they test more than 80 patients with mCRC per year, and 2.1 % (*n* = 2) estimated testing fewer than 20 patients per year. A full description of the participating laboratories is given in Table 1.

**Table 1:** Description of participating pathology laboratories

Variable (*n*)	Criterion	Frequency	%
Estimated number of patients with mCRC tested per year (*n* = 96)	>80	69	71.9
	≤80	27	28.1
Reported indication for *RAS* mutation testing (*n* = 95)	“On request from an oncologist”	85	89.5
	“All CRC patients tested”	5	5.3
	“Other”^a^	5	5.3
Location of *RAS* mutation testing (*n = 96)*	Own institution	90	93.8
	External	1	1.0
	Own institution and external	5	5.2
Minimum percentage of neoplastic cells required (*n* = 96)	No cut-off defined	10	10.4
	<10 %	18	18.8
	≥10 %	68	70.8
DNA extraction method used (*n* = 96)	QIAamp DNA FFPE kit (Qiagen)	40	41.7
	Cobas DNA Sample Preparation kit (Roche)	12	12.5
	QIAamp DNA mini kit (Qiagen)	7	7.3
	Raw proteinase K lysate	6	6.3
	Maxwell 16 (Promega)	14	14.6
	MagNA Pure (Roche)	1	1.0
	Other	16	16.7
*RAS* mutations tested (*n* = 96)	All codons tested	70	72.9
	Not all codons tested	26	27.1

^a^“Other” reported indications for *RAS* testing were: “All stage III & IV CRC patients are tested”, “In our hospital, all CRC patients are tested. Referrals from other centres are tested on demand from the oncologist”, “Diagnostic combination”, “On request from an oncologist as well as in known metastatic (M1) CRC patients” and “Requested by oncologist and pathologist”. *CRC* colorectal cancer, *mCRC* metastatic CRC

### *RAS* testing methods

The majority of participating institutions (89.5 %, *n* = 85) reported that they carry out *RAS* testing only “On request from an oncologist”, whereas 5.3 % (*n* = 5) of laboratories reported testing “All patients with CRC” and 5.3 % (*n* = 5) cited “Other” indications. *RAS* testing was most frequently performed onsite within the reporting institution (93.8 %, *n* = 90); 5.2 % (*n* = 5) of respondents reported a mixture of both onsite and external (offsite) testing. A single respondent reported only external testing of tumour samples for *RAS* mutation status (Table 1).

Overall, 89.6 % (*n* = 86) of laboratories reported that they use a minimum cut-off percentage of neoplastic cells for histopathological assessment and subsequent *RAS* testing. For the 86 laboratories using a cut-off value, the reported minimum percentage of neoplastic cells ranged from 1 to 50 %, with 18.8 % (*n* = 18) of the laboratories reporting their minimum cut-off for testing at <10 % and 70.8 % (*n* = 68) at ≥10 % (mean: 14.9 %; median: 10.0 %) (Table 1).

There were five main DNA extraction methods used by at least one of the laboratories surveyed, of which the QIAamp DNA FFPE kit (Qiagen) (41.7 %), the Maxwell 16 system (Promega) (14.6 %) and the Cobas DNA Sample Preparation kit (Roche) (12.5 %) were the most commonly used (Table 1).

All 96 survey respondents reported testing for *KRAS* exon 2 mutations. The implementation of testing for the other *RAS* mutations (in *KRAS* exons 3 and 4, and *NRAS* exons 2, 3 and 4) varied from 76.0 to 95.8 %. The majority (72.9 %, *n* = 70) of the survey respondents reported testing all 12 relevant codons. Three (3.1 %) participants reported only testing *KRAS* exon 2. Full details of the rate of *RAS* mutation testing by *RAS* codon are shown in Table 2. Testing of extracted DNA for *RAS* mutation status was assessed on a by-codon basis and the responses divided into either those that used commercially available CE-IVD kits or those using sequencing-based methods. Overall no clear preference in DNA testing method was observed, but CE-IVD kits were most often used for testing *KRAS* exon 2, codons 12 and 13, by 47 and 48 % of respondents, respectively, compared with 30 % of participants using sequencing-based techniques for both codons. The same testing method was used for all codons by 68.8 % of the respondents. Pathology centres reported using the Therascreen *KRAS*/*NRAS* pyro kit (Qiagen) most often, but with frequencies varying from 8 to 14 % depending on the codon being tested. The second most frequently used kit was the *KRAS*/*NRAS* mutation detection kit (EntroGen). For those laboratories using sequencing-based methods, the most commonly used technique across all codons was dideoxy (Sanger) sequencing (non-proprietary) ranging from 15 to 26 % of respondents depending on which codon was being tested. The second and third most frequently used sequencing-based methods were Ion AmpliSeq (Life Technologies) and Pyrosequencing (Qiagen), respectively, with use by respondents reported as ranging from 7 to 9 % and 1 to 6 %, respectively, again depending on the tested codon.

**Table 2:** Frequency and percentage of laboratories using CE-IVD kits and sequencing-based methods by *RAS* codon

Exon	*KRAS* exon 2		*KRAS* exon 3		*KRAS* exon 4		*NRAS* exon 2		*NRAS* exon 3		*NRAS* exon 4	
Codon	12	13	59	61	117	146	12	13	59	61	117	146
Total laboratories testing, *n* (%)	*96 (100)*	*96 (100)*	*78 (81)*	*92 (96)*	*86 (90)*	*87 (91)*	*90 (94)*	*90 (94)*	*79 (82)*	*90 (94)*	*73 (76)*	*80 (83)*
CE-IVD kit (commercial kit), *n* (%)	45 (47)	46 (48)	26 (27)	40 (42)	31 (32)	31 (32)	35 (37)	35 (37)	27 (28)	35 (37)	26 (27)	32 (33)
Cobas *KRAS* mutation test (Roche)	11 (12)	11 (12)	3 (3)	11 (12)	1 (1)	1 (1)	1 (1)	1 (1)	1 (1)	1 (1)	1 (1)	1 (1)
Therascreen *KRAS/NRAS* pyro kit (Qiagen)	9 (9)	10 (10)	10 (10)	11 (12)	9 (9)	9 (9)	13 (14)	13 (14)	9 (9)	13 (14)	8 (8)	9 (9)
*KRAS/NRAS* mutation detection kit (EntroGen)	10 (10)	9 (9)	3 (3)	10 (10)	9 (9)	9 (9)	10 (10)	10 (10)	4 (4)	10 (10)	5 (5)	10 (10)
Therascreen *KRAS* RGQ PCR kit (Qiagen)	5 (5)	6 (6)	0 (0)	1 (1)	0 (0)	0 (0)	0 (0)	0 (0)	0 (0)	0 (0)	0 (0)	0 (0)
*KRAS/NRAS* StripAssay (ViennaLab)	2 (2)	2 (2)	0 (0)	1 (1)	0 (0)	0 (0)	2 (2)	2 (2)	0 (0)	2 (2)	0 (0)	0 (0)
Anti-EGFR MoAb response *KRAS/NRAS* (Diatech)	3 (3)	3 (3)	3 (3)	3 (3)	3 (3)	3 (3)	3 (3)	3 (3)	3 (3)	3 (3)	3 (3)	3 (3)
Therascreen *KRAS* PCR kit (Qiagen)	1 (1)	1 (1)	0 (0)	0 (0)	0 (0)	0 (0)	0 (0)	0 (0)	0 (0)	0 (0)	0 (0)	0 (0)
*KRAS/NRAS* LightMix (TIB Molbiol)	1 (1)	1 (1)	0 (0)	0 (0)	3 (3)	3 (3)	3 (3)	3 (3)	3 (3)	3 (3)	3 (3)	3 (3)
*RAS* extension pyro kit (Qiagen)	2 (2)	2 (2)	6 (6)	2 (2)	5 (5)	5 (5)	2 (2)	2 (2)	6 (6)	2 (2)	5 (5)	5 (5)
*KRAS/NRAS* gene mutation detection kit (Diatech)	1 (1)	1 (1)	1 (1)	1 (1)	1 (1)	1 (1)	1 (1)	1 (1)	1 (1)	1 (1)	1 (1)	1 (1)
PCR+sequencing or sequencing, *n* (%)	29 (30)	29 (30)	35 (37)	33 (34)	38 (40)	39 (41)	37 (39)	37 (39)	35 (37)	38 (40)	33 (34)	34 (35)
Dideoxy (Sanger) sequencing	14 (15)	14 (15)	20 (21)	18 (19)	24 (25)	25 (26)	22 (23)	22 (23)	21 (22)	22 (23)	25 (26)	25 (26)
Pyrosequencing (Qiagen)	5 (5)	5 (5)	5 (5)	5 (5)	4 (4)	4 (4)	5 (5)	5 (5)	4 (4)	6 (6)	1 (1)	2 (2)
Ion AmpliSeq - Ion Torrent (Life Technologies)	9 (9)	9 (9)	9 (9)	9 (9)	9 (9)	9 (9)	9 (9)	9 (9)	9 (9)	9 (9)	7 (7)	7 (7)
The TruSeq Amplicon - Cancer Panel (Illumina)	1 (1)	1 (1)	1 (1)	1 (1)	1 (1)	1 (1)	1 (1)	1 (1)	1 (1)	1 (1)	0 (0)	0 (0)
Other methods, *n* (%)	16 (17)	15 (16)	10 (10)	10 (10)	10 (10)	9 (9)	12 (13)	12 (13)	11 (11)	11 (11)	8 (8)	8 (8)
Multiple methods, *n* (%)	6 (6)	6 (6)	7 (7)	9 (9)	7 (7)	8 (8)	6 (6)	6 (6)	6 (6)	6 (6)	6 (6)	6 (6)

*PCR* polymerase chain reaction

Comprehensive details of the *RAS* testing methods that were used by the participating laboratories for each codon are shown in Table 2.

### *RAS* mutation prevalence

Of the 3,259 patients included in the aggregated data, 3,244 (99.5 %), for whom *RAS* status was known and documented, were included in the subsequent *RAS* mutation prevalence analysis. The overall *RAS* mutation prevalence was 46.0 % (95 % CI: 44.3–47.7 %) for all included patients. In a subgroup of 2,245 (68.9 %) patients for whom all *RAS* hot-spot codons were tested, the total *RAS* mutation prevalence was 48.5 % (95 % CI: 46.4–50.6 %). All subsequent *RAS* mutation prevalence analyses and results described were restricted to this subgroup.

There was no significant variation in the rates of *RAS* mutation prevalence by country (*P* = 0.461) for those countries with at least three participating laboratories (excluding any laboratories that did not test all codons). Country-specific *RAS* mutation prevalence ranged from 40.0 % (95 % CI: 31.2–49.3 %) in Belgium to 52.1 % (95 % CI: 44.7–59.5 %) in France.

**Fig. 2:**
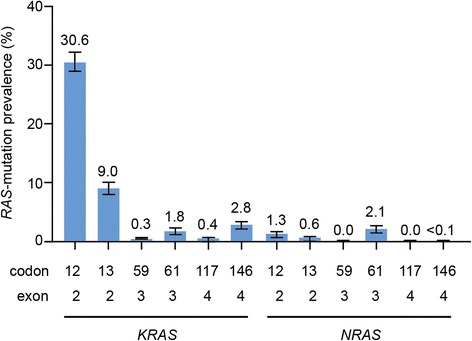
*RAS* mutation prevalence by codon for tumour samples tested for all *RAS* codons (*n* = 2,245)

The highest rates of *RAS* mutation prevalence for laboratories that tested all *RAS* hot-spot codons were reported for *KRAS* exon 2, codons 12 and 13: 30.6 % (95 % CI: 28.7–32.5 %) and 9.0 % (95 % CI: 7.9–10.3 %), respectively. For the other codons, mutation rates ranged from <0.1 to 2.8 %, with the exception of *NRAS* exon 3, codon 59 and *NRAS* exon 4, codon 117, for which no patients were identified with these *RAS* mutations (Fig. 2). In this cohort, mutations affecting *KRAS* exon 2, codons 12 and 13 accounted for 62 and 18 %, respectively, of all *RAS* mutations identified.

For the 1,393 (42.7 %) patients with a documented primary CRC tumour site, *RAS* mutation prevalence was found to vary significantly by location when comparing right and left colon primary tumours: 54.6 % (95 % CI: 50.2–59.0 %) and 46.4 % (95 % CI: 41.6–51.2 %), respectively (*P* = 0.012). However, when comparing right and left colon cancers with rectal tumours, for which the *RAS* mutation prevalence was 51.0 % (95 % CI: 46.3–55.7 %), there was no overall significant difference (*P* = 0.043). There was also no significant difference in *RAS* mutation prevalence according to the type of tissue sample used for *RAS* testing, categorised as either primary or secondary (metastatic) tumour tissue (Table 3).

**Table 3:** *RAS* mutation prevalence estimates for tumour samples tested for all *RAS* codons

		*RAS* mutation status		*RAS* mutation prevalence		
Variable (*n*)	Criterion	Wild-type	Mutated	(%)	95 % CI	*P*-value
Overall *RAS* mutation prevalence (*n* = 2,245)	Patients with all codons tested only	1,156	1,089	48.5	(46.4–50.6)	
Location of primary tumour^a^ (*n* = 1,393)	Right colon (proximal to splenic flexure)	232	279	54.6	(50.2–59.0)	
	Left colon (distal to splenic flexure)	230	199	46.4	(41.6–51.2)	0.012^b^
	Rectum	222	231	51.0	(46.3–55.7)	0.043^c^
Tissue type isolated^a^ (*n* = 1,669)	Primary tumour	651	653	50.1	(47.3–52.8)	
	Metastatic site	184	181	49.6	(44.3–54.8)	0.869
Number of patients tested per year (*n* = 2,093)	>80	861	850	49.7	(47.3–52.0)	
	≤80	295	239	44.8	(40.5–49.0)	<0.001
Indication for testing (*n* = 2,215)	“On request from an oncologist”	1,019	964	48.6	(46.4–50.8)	
	“All patients with CRC tested”	33	51	60.7	(49.5–71.2)	
	“Other”	84	64	43.2	(35.1–51.6)	0.036^d^
Location of testing (*n* = 2,245)	Own institution	1,117	1,054	48.5	(46.4–50.7)	
	Own institution and external	39	35	47.3	(35.6–59.3)	0.832
Minimum percentage of neoplastic cells (*n* = 2,445)	No cut-off defined	75	78	51.0	(42.8–59.1)	
	Cut-off defined	1,081	1,011	48.3	(46.2–50.5)	0.526
Cut-off percentage of neoplastic cells (*n* = 2,092)	Cut-off <10 %	177	137	43.6	(38.1–49.3)	
	Cut-off ≥10 %	904	874	49.2	(46.8–51.5)	0.071
DNA extraction method used (*n* = 2,245)	QIAamp DNA FFPE kit (Qiagen)	475	463	49.4	(46.1–52.6)	
	Cobas DNA Sample Preparation kit (Roche)	75	73	49.3	(41.0–57.7)	
	QIAamp DNA mini kit (Qiagen)	79	56	41.5	(33.1–50.3)	
	Raw proteinase K lysate	97	79	44.9	(37.4–52.6)	
	Maxwell 16 (Promega)	192	178	48.1	(42.9–53.3)	
	MagNAPure (Roche)	15	19	55.9	(37.9–72.8)	
	Other	223	221	49.8	(45.0–54.5)	0.550

^a^Only includes wild-type and mutated results. Patients with unknown/unavailable *RAS* mutation status have been excluded

^b^Comparison of *RAS* mutation prevalence between right colon and left colon primary tumours only, excluding data from rectal tumours

^c^Comparison of *RAS* mutation prevalence between right colon, left colon and rectal primary tumours

^d^For the purposes of comparing *RAS* mutation prevalence, patients reported as having been tested due to “Other” indications have been grouped together

Of note, patients reported in aggregated data sample may have had *RAS*-family mutations affecting more than one oncogene

*CRC* colorectal cancer, *CI* confidence interval

The *RAS* mutation prevalence calculated for laboratories that estimated testing >80 patients with mCRC for *RAS* status each year was significantly higher than for laboratories that estimated testing ≤80 patients: 49.7 % (95 % CI: 47.3–52.0 %) compared with 44.8 % (95 % CI: 40.5–49.0 %). *RAS* mutation prevalence also varied significantly according to the indication given for testing: 60.7 % (95 % CI: 49.5–71.2 %) for *“*All CRC patients are tested” compared with 48.6 % (95 % CI: 46.4–50.8 %) for *“*On request from an oncologist”, and 43.2 % (95 % CI: 35.1–51.6 %) for “Other” indications (Table 3).

There were no significant differences in *RAS* mutation prevalence when comparing onsite with offsite testing, DNA extraction method used and whether or not laboratories used a cut-off for the minimum percentage of neoplastic cells, or if the cut-off was <10 % or ≥10 % (Table 3).

### *RAS* testing turnaround time

Overall, for the 3,171 (97.3 %) patients with CRC for whom turnaround time was documented, results were reported back to the requesting physician in ≤5 working days after the test was requested in nearly half of the cases (47.1 %). Only 9.2 % of *RAS* testing results had a reported turnaround time of >10 working days.

Reported turnaround times varied for each country, with Switzerland, Austria and Denmark having the greatest proportions of patients with a turnaround time of ≤5 working days: 85.3 %, 75.6 % and 64.2 %, respectively (*P* < 0.001). By contrast, Turkey, the Czech Republic and Sweden had the greatest proportions of patients with a documented turnaround time of >5 working days: 100 %, 95.6 % and 92.7 %, respectively (*P* < 0.001).

Laboratories that estimated the number of patients with mCRC tested for *RAS* mutation status per year as >80 had longer turnaround times compared with those that estimated testing ≤80 patients per year: 40.0 % vs. 61.0 % in ≤5 days, respectively (*P* < 0.001). A comparison of turnaround times for patients according to which *RAS* codons had been tested, demonstrated that turnaround times were ≤5 days for 44.4 % of those tested for all codons and 54.1 % for patients with only partial *RAS* mutation testing (*P* < 0.001). Laboratories using the same *RAS* mutation testing method for all codons being tested had shorter turnaround times than those in which more than one method was used: 50.1 % vs. 32.7 % in ≤5 days, respectively (*P* < 0.001).

Reported turnaround times also varied according to the clinical indication given for *RAS* testing. For patients tested at the request of an oncologist, and patients tested at institutions that test all patients with CRC, the proportions with a turnaround time of ≤5 days were 46.2 and 32.1 %, respectively. For patients tested at institutions that reported other indications for *RAS* testing, the proportion with a turnaround time of ≤5 days was 74.2 %. Laboratories that reported carrying out *RAS* testing at their own institution had shorter turnaround times compared with those that reported using a mixture of onsite and external testing: 48.8 % vs. 11.7 % of results were reported in ≤5 days, respectively (*P* < 0.001). The aggregated patient data by turnaround time are shown in detail in Table 4.

**Table 4:** Turnaround time for *RAS* testing results by country and testing practices

		Turnaround time (working days)		
Variable (*n*)	Criterion	≤5 (%)	6–10 (%)	>10 (%)
*n* = 3,191		1,511 (47.4)	1,389 (43.5)	291 (9.1)
Country^a^ *n* = 3,171	Austria (*n* = 201)	152 (75.6)	49 (24.4)	0 (0.0)
	Belgium (*n* = 240)	70 (29.2)	93 (38.8)	77 (32.1)
	Czech Republic (*n* = 90)	4 (4.4)	65 (72.2)	21 (23.3)
	Denmark (*n* = 120)	77 (64.2)	39 (32.5)	4 (3.3)
	France (*n* = 238)	60 (25.2)	111 (46.6)	67 (28.2)
	Italy (*n* = 276)	109 (39.5)	149 (54.0)	18 (6.5)
	Netherlands (*n* = 457)	259 (56.7)	194 (42.5)	4 (0.9)
	Norway (*n* = 74)	29 (39.2)	27 (36.5)	18 (24.3)
	Poland (*n* = 80)	43 (53.8)	32 (40.0)	5 (6.3)
	Spain (*n* = 192)	55 (28.7)	134 (69.8)	3 (1.6)
	Sweden (*n* = 82)	6 (7.3)	72 (87.8)	4 (4.9)
	Switzerland (*n* = 415)	354 (85.3)	56 (13.5)	5 (1.2)
	Turkey (*n* = 90)	0 (0.0)	66 (73.3)	24 (26.7)
Number of patients tested per year (*n* = 3,191)	>80	828 (40.0)	1,022 (49.3)	222 (10.7)
	≤80	683 (61.0)	367 (32.8)	69 (6.2)
*RAS* mutations tested (*n* = 3,191)	All codons tested	983 (44.4)	1,102 (49.8)	130 (5.9)
	Not all codons tested	528 (54.1)	287 (29.4)	161 (16.5)
Same testing method for all codons (*n* = 3,191)	Yes	1,345 (50.1)	1,142 (42.6)	197 (7.3)
	No	166 (32.7)	247 (48.7)	94 (18.5)
Indication for *RAS* testing (*n* = 3,161)	“On request from an oncologist”	1,325 (46.2)	1,288 (44.9)	258 (9.0)
	“All CRC patients tested”	36 (32.1)	56 (50.0)	20 (17.9)
	“Other”	132 (74.2)	33 (18.5)	13 (7.3)
Location of testing (*n* = 3,191)	Own institution	1,496 (48.8)	1,343 (43.9)	224 (7.3)
	Own institution and external	15 (11.7)	46 (35.9)	67 (52.3)

^a^Countries with fewer than three laboratories have been excluded from this table

## Discussion

Recent revisions to the prescribing guidelines for anti-EGFR mAbs require *RAS* genotyping in patients with mCRC prior to the initiation of therapy. These revisions have necessitated a change in the management and testing of patients with mCRC, and thus highlight the need for investigation into *RAS* mutation testing practices and their variability within Europe.

Here we report results from an online survey of 96 pathology laboratories from 24 European countries. All 96 laboratories reported testing for *KRAS* exon 2 mutations, and the majority (72.9 %) reported testing all the required *RAS* codons as standard. The findings of this survey confirm the increase in implementation of *RAS* mutation testing that has been reported in recent studies both within and outside of Europe [20, 21]. Results of the 2013 ESP Colon EQA scheme, which included 131 laboratories from 30 different countries, showed that 49.3 % of the participating laboratories had implemented *RAS* testing for all hot-spot codons [20]. A number of factors may have contributed to the disparity in the proportions of laboratories reportedly testing all *KRAS* and *NRAS* codons between this survey and the 2013 EQA scheme; in particular, the latter was initiated very soon after the revisions to the EMA indications for anti-EGFR mAbs, and included participants from outside of Europe. Fewer than half of the participating laboratories were accredited by a NAB, although the response rate was higher among these institutions than among non-accredited laboratories. This is in agreement with reports from the ESP EQA scheme, which observed that few laboratories participating have been accredited according to a well-known international standard [20]. This highlights the need for increased efforts to encourage more laboratories to seek accreditation.

In the present survey we found that the majority of laboratories (71.9 %) test >80 patients a year for *RAS* mutation status, with testing typically carried out at the requesting institution (93.8 %) and at the request of an oncologist (89.5 %). Only 5.3 % of laboratories routinely test all their patients with CRC for *RAS* mutation status; however, this means that the information is immediately available to the treating oncologists at these institutions prior to considering treatment with anti-EGFR mAbs. *RAS* mutation testing methodologies vary considerably among pathology laboratories and according to the codon being tested. Overall the reported use of different categories of testing methods was broadly similar to that of previous ESP EQA schemes [20, 22]. Our findings not only confirm that dideoxy sequencing remains the single most commonly used method, but also that the use of next-generation sequencing techniques and of commercially available kits, such as the Cobas *KRAS* mutation test (Roche) and the Therascreen *KRAS/NRAS* pyro kit, has remained consistent over the last 3 years. The high degree of variability in *RAS* testing methods used among different laboratories underscores the need for EQA schemes to assess and ensure the ongoing accuracy and precision of *RAS* mutation testing.

The overall crude *RAS* mutation prevalence was calculated as 48.5 % (95 % CI: 46.4–50.6 %) for patients tested for all relevant *RAS* codons. The calculated overall *RAS* mutation prevalence in this study was consistent with findings from sequenced CRC tumours in the 2012 TCGA database (49 %) and from a recent study of the reproducibility of *RAS* testing among pathology centres in the Netherlands (47.6 %), but was slightly lower than in a recently published pooled analysis of clinical trials of anti-EGFR therapy in patients with mCRC, which showed an overall *RAS* mutation prevalence of 55.9 % (95 % CI: 53.9–57.9 %) [23–25]. *RAS* mutation prevalence estimates varied significantly by country, approximate number of patients tested per year and the indication for *RAS* testing and between left- and right-sided tumours. Previous research has indicated that *RAS* mutated tumours occur more frequently in the ascending (right) colon than the descending (left) colon [26–28]. The results from the present survey support this conclusion, showing that the prevalence of *RAS* mutations was higher in patients with right-sided primary tumours compared with those with left-sided primary tumours. The *RAS* mutation prevalence observed at centres that routinely tested all patients with CRC appeared unusually high when compared with the overall prevalence rate in this study. However, it is important to note that the sample size for this subgroup was small (five pathology centres providing data for 84 patients). Therefore, this result needs to be interpreted with caution.

Turnaround time was found to be ≤10 working days, which is recommended for routine clinical practice for the majority of patients (90.8 %). However, nearly half (47.1 %) of the patients assessed had their result reported in ≤5 days. It should be noted that, as turnaround time was defined as the time from the laboratory receiving the request to reporting of the result back to the requesting physician, the real time may be longer in some cases, for example due to transportation of tissue blocks from one laboratory to another. Factors that prolonged turnaround time were testing of >80 patients a year (which may be due to overburdening of laboratories), testing of all *RAS* codons and external testing of some patient samples. When considering therapy with anti-EGFR mAbs it is important that the *RAS* testing results are made available to the requesting oncologist as quickly as possible as patients with mCRC can deteriorate rapidly, over a period of weeks, and need urgent, effective, treatment decisions.

Although the overall response rate (49.5 %) for this study was relatively high for an online survey, it may not be fully representative of European laboratory practices. The survey was intended to be completed by the molecular biologist responsible for molecular diagnostics at each of the participating laboratories, however this could not be verified from the survey results, and it is possible that in some instances it was completed by a technician or another laboratory representative.

Determining *RAS* mutation prevalence and variation on the basis of aggregated patient CRC data is a potential limitation of this study, as it was not possible to account for the influence of non-reported patient-specific factors and clinical variables that may have influenced the results. Also, because certain clinical findings are often omitted from pathology records, data for some of the categories were not available for a large proportion of the patients. Finally, recent clinical guidelines have recommended the use of resected tissues for *RAS* mutation testing, where possible, rather than biopsy specimens [29], but information about the type of tissue used could not be captured in the present study. Furthermore, although it is reasonable to assume that most samples have been taken from patients with mCRC, it is likely that a small proportion of tumour samples will have been collected (by laboratories routinely testing all CRC patients) from patients who did not have any evidence of metastases at the time. Therefore the data presented may not exclusively represent a population of mCRC patients. However, it has been shown previously that there is a high concordance of *KRAS* exon 2 mutation status between primary colorectal tumours and their corresponding liver metastases [30].

## Conclusions

The findings from this study show that implementation of full *RAS* testing, for exons 2, 3 and 4 of *KRAS* and *NRAS*, is high but not yet universal, with nearly three-quarters of the participating laboratories reporting full testing of the relevant *RAS* oncogenes. This would seem to reflect an overall upward trend in the implementation of full *RAS* testing, with the rate documented in this study considerably higher than the 49.3 % of laboratories testing all codons as reported in the results from the 2013 ESP Colon EQA scheme [20]. A small minority of the respondents (*n* = 3) reported that they still only test *KRAS* exon 2 (the previous EMA indication for the use of anti-EGFR mAbs).

This is the first study to capture turnaround time for *RAS* testing, and our findings showed that the turnaround time for results is ≤5 working days for almost half of the laboratories that participated. Further observational studies will be needed to clarify whether the implementation and standardisation of *RAS* mutation testing changes significantly in the near future. However, these findings, showing current variation of *RAS* testing practices, contribute to the developing body of evidence relating to the prevalence of *RAS* mutations and create awareness of factors that can affect turnaround time and accurate detection of all *RAS* mutations.

## Abbreviations

CI: Confidence interval
CRC: Colorectal cancer
EGFR: Epidermal growth factor receptor
EMA: European Medicines Agency
EQA: External quality assurance
ESP: European Society of Pathology
*KRAS*: Kirsten rat sarcoma
mAb: Monoclonal antibody
mCRC: Metastatic colorectal cancer
NAB: National accreditation body
*NRAS*: Neuroblastoma rat sarcoma
*RAS*: Rat sarcoma
